# The BTLA–HVEM–CD5 Immunoregulatory Axis–An Instructive Mechanism Governing pTreg Cell Differentiation

**DOI:** 10.3389/fimmu.2019.01163

**Published:** 2019-05-22

**Authors:** Jessica Bourque, Daniel Hawiger

**Affiliations:** Department of Molecular Microbiology and Immunology, Saint Louis University School of Medicine, St. Louis, MO, United States

**Keywords:** BTLA, HVEM, CD5, pTreg cells, dendritic cells

## CD5—a Bridge Between Thymic Selection and Peripheral Differentiation of Treg Cells

CD5 is a cysteine-rich scavenger-like receptor expressed by B-1a and T cells that is generally thought to be a negative regulator of antigen-specific receptor signaling (1). In developing thymocytes, expression of CD5 correlates with T cell receptor (TCR) signal strength, resulting in high CD5 expression by thymocytes that strongly interact with self-peptide: major histocompatibility complexes (MHC) (2). While the mechanisms that mediate the ability of CD5 to dampen TCR signaling are still partially unclear, it has been shown that negative regulators, including SHP-1, Ras-GAP, c-Cbl and casein kinase 2 (CK2), are recruited to the cytoplasmic domain of CD5 (1, 3). Further, CD5 has been shown to interact with TCR signaling molecules such as PI3K, Fyn, Lck, and ZAP-70 (1, 4, 5). Other functions of CD5 in mature peripheral T cells influence their survival, anergy, and T helper 17 (Th17) cell differentiation. These functions of CD5 rely on regulation of the activation of mechanistic target of rapamycin (mTOR) as well as crucial interactions between CD5 and CK2 (3, 6, 7). Although ligation of CD5 can modify its functions, the cell-autonomous functions of CD5 in T cells are independent of CD5 engagement of its extracellular domain by a specific ligand (8). In contrast, such cell-autonomous functions of CD5 depend on its specific level of expression. In the thymus, negative regulation of TCR signaling by CD5 allows for strongly self-reactive thymocytes to escape deletion during thymic selection, therefore extending the range of naïve T cells cross-reactive against various pathogens (2, 9–14). These T cells retain high expression of CD5 upon their migration to the periphery, so the expression of CD5 in T cells can be used as a marker of TCR signal strength (1). Further, a specific high or low expression of CD5 may also indirectly mark some T cells differing in intrinsic changes in TCR signaling pre-determined by TCR interactions in the thymus (14, 15). Overall, the increased expression of CD5 in CD4^+^ T cells may serve as an indicator of self-reactivity in the polyclonal T cell repertoire and such CD5^hi^ T cells present greater risks for autoimmune responses (11–14).

Additional specific mechanisms are therefore needed to mitigate the risk of autoimmune responses by self-reactive CD5^hi^ T cells that are released into the periphery. Our previous results elucidated a CD5-dependent mechanism, separate from its role in regulation of TCR signaling, that facilitates the formation of peripheral regulatory T (pTreg) cells from CD5^hi^ T cells (16, 17). This mechanism is also consistent with the previously proposed idea of precursors for pTreg cells that are present among peripheral T cells (18, 19). In contrast to the CD5-dependent process of pTreg cell conversion, the development of tTreg cells in thymus is independent of CD5 functions (20, 21). We found that CD5 promotes induction of Foxp3 expression and conversion into pTreg cells by opposing in CD5^hi^ T cells the activation of mTOR mediated by effector differentiating cytokines such as interleukin-4 (IL-4), IL-6, and interferon-γ (IFN-γ) that can be constantly produced by small numbers of effector T cells present even under physiological steady state conditions (16, 17). Similarly *in vitro*, the functions of CD5 prevent effector differentiating cytokines from blocking the TGF-β mediated induction of iTreg cells (17, 22). This effect on mTOR activation is likely mediated through CD5 interference with PI3K signaling, and an inhibition of either PI3K or mTOR leads to a restored conversion of Treg cells in T cells lacking CD5 functions (17). This is consistent with the established roles of PI3K and mTOR in the inhibition of Treg cell differentiation (23–25). While it is still unclear how CD5 inhibits PI3K/AKT/mTOR signaling in pTreg cells, CK2 may be involved in this process as it has been shown to modulate activation of this pathway in other contexts (26, 27). Further, it remains unclear to what extent the initially formed CD5^hi^ T cells can resist subsequent differentiation into effector T cells under acute pro-inflammatory conditions. Future research may clarify this.

## Regulation of Peripheral CD5 Expression by Dendritic Cells

**Figure 1:**
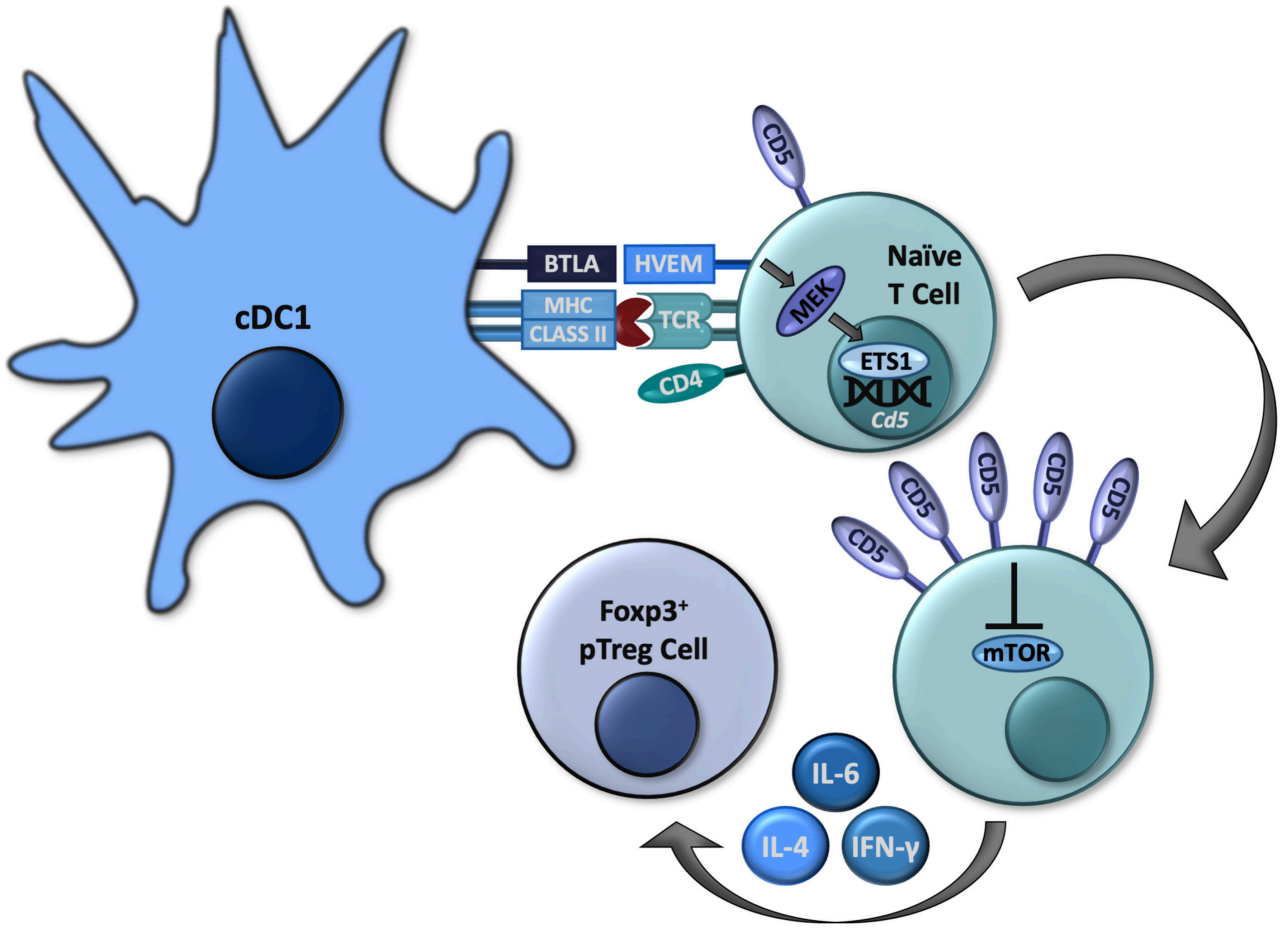
Some cDC1s express BTLA, which can signal through HVEM in naïve CD4^+^ T cells to activate MEK and subsequently ETS1 to increase expression of *Cd5*. High CD5 expression allows these to cells to convert to Foxp3^+^ pTreg cells by interfering with mTOR activation, even in the presence of effector-differentiating cytokines such as IL-4, IL-6, and IFN-γ.

The formation of pTreg cells is crucial for peripheral tolerance and prevention of specific autoimmunity (28, 29). However, as discussed above, the expression of CD5 among a polyclonal T cell repertoire represents a spectrum resulting in different expression of CD5 in T cells of various antigenic specificities (11–14). Therefore, the designation of T cells leaving the thymus as “CD5^hi^” is in fact relative. Also, despite a lower affinity for the peptides represented in thymus, some of the developing CD5^lo^ T cells can still include clones highly responsive to peripheral self-antigens (30). Therefore, the increased expression of CD5 induced in the thymus cannot sufficiently direct a conversion of antigen-specific pTreg cells. Further, a model that relies on pTreg cell conversion being pre-programmed in the thymus is not easily reconciled with the well-established instructive roles of peripheral dendritic cells (DCs) to govern pTreg cell conversion in response to specific antigens and other signals perceived by such T cells and DCs in the periphery (28). However, in addition to the mechanisms regulating CD5 expression previously identified in the thymus, peripheral T cells can also increase their CD5 expression in response to pro-tolerogenic stimulation (31, 32). Such upregulation of CD5 expression in peripheral self-reactive T cells may therefore act to further promote a conversion into pTreg cells. We recently clarified the mechanisms governing induction of CD5 expression in peripheral T cells. Results from our laboratory have elucidated crucial roles for the immunoregulatory axis dependent on the immunoglobulin superfamily member B and T lymphocyte associated (BTLA) expressed on some DCs (BTLA^hi^) that signals through herpesvirus entry mediator (HVEM) in T cells to up regulate the expression of CD5 and instruct the conversion of pTreg cells under physiological conditions that include multiple pro-effector and pro-tolerogenic cytokines (17, 22) and (Figure 1).

DCs are antigen presenting cells (APCs) that are critical for the initiation and regulation of T cell responses to foreign and self-antigens. The outcomes of antigen-specific interactions between DCs and T cells are governed by immunomodulatory molecules expressed by each cell type (33). Both human and murine DCs consist of two main populations, conventional (myeloid) (cDC) and plasmacytoid (pDC), both of which develop from progenitors in the bone marrow that differentiate into various subsets present throughout multiple tissues (34–36). The cDC population can be further divided into the cDC1 and cDC2 subsets, defined by the transcription factors required for their development. The cDC1 subset, which requires the transcription factors Irf8, Id2, and Batf3 for development, can be distinguished by expression of XCR1 and BTLA. In contrast, the cDC2 subset depends on the transcription factor Irf4 for development and is distinguished by expression of CD172a (SIRPα) (22, 34, 35). In the absence of specific acute pro-inflammatory stimuli (steady state), DCs generally promote T cell tolerance that crucially relies on the induction of pTreg cells (29, 37, 38). Although the developmental designation of DC subsets does not strictly overlap with their distinct immune functions, the specific subsets are characterized by a degree of functional specialization. Whereas, cDC2s can preferentially promote Th2, Th17, and follicular helper T cell differentiation, cDC1s have crucial roles for the cross-priming of CD8^+^ T cells, priming of Th1 cells, and induction of CD4^+^CD25^+^Foxp3^+^ pTreg cells (28, 33, 34, 39).

## BTLA and HVEM Instructed Regulation of CD5 Expression

In addition to cDC1s, BTLA is also expressed in T cells, B cells, macrophages, NK cells, and NKT cells (22, 40–42). BTLA contains three immunoreceptor tyrosine-based inhibition motifs (ITIMs) which, upon phosphorylation, recruit Src homology domain 2 (SH2)-containing protein tyrosine phosphatases, SHP-1 and SHP-2, which exert a variety of inhibitory effects within various lymphocytes (40, 41). However, in addition to the above-described intrinsic signaling mediated by its intracellular domains, BTLA also serves as a ligand for HVEM, a tumor necrosis factor receptor (TNFR) superfamily member primarily expressed in naïve CD4^+^ and CD8^+^ T cells, which downregulate its expression following activation (41). HVEM contains four extracellular cysteine-rich domains (CRDs) that mediate its specific binding to BTLA or other ligands such as LIGHT in different conformations (41). Following ligation by LIGHT, HVEM activates PI3K leading to immunogenic lymphocyte activation (43). Upon binding to BTLA, HVEM can signal through TNF receptor associated factor 2 (TRAF2) to induce phosphorylation of STAT3, resulting in NF-kB activation as well as other pro-survival signals (44, 45). These interactions between BTLA and HVEM can also modulate a variety of immunological processes, including CD8^+^ T cell survival and memory formation, Treg cell functionality, and DC homeostasis (41, 42, 45, 46). We recently established specific roles for BTLA and HVEM in governing a conversion of pTreg cells through the modulation of CD5 expression in T cells (22). We found that in antigen-specific T cells activated by BTLA-expressing cDC1s, HVEM-mediated signals lead to an increased phosphorylation of mitogen-activated protein kinase (MAPK) kinase (MEK) and expression of ETS1, resulting in increased transcription of *Cd5* and the corresponding increased surface expression in CD5^hi^ T cells (22). While other signals might contribute to regulation of CD5 expression, BTLA-HVEM signaling is indispensable for the upregulation of CD5 in T cells activated by DCs *in vivo* under normal physiological conditions (22). The subsequent conversion of such DC-induced CD5^hi^ T cells into pTreg cells is then enhanced because CD5^hi^ T cells become resistant to the specific mTOR-dependent signals mediated by effector differentiating cytokines as discussed above (Figure 1).

## Conclusions and Future Directions

The regulation of immune tolerance by DCs through the induction of pTreg cells is mediated by various mechanisms utilized by specific subsets of DCs (28, 33). The BTLA-HVEM-CD5 signaling axis is critically important for the ability of cDC1s to promote differentiation of pTreg cells that have crucial functions in blocking an autoimmune process (22). Despite the finding that BTLA-HVEM signaling upregulates CD5 expression in T cells, the mechanisms utilized by CD5 to modulate functions of mTOR and to promote the differentiation of pTreg cells still need to be fully clarified. Particularly, determining the possible roles of the established interactions between CD5 and CK2 could help to elucidate this process. Further, CD5 may govern relevant molecular functions in the newly formed pTreg cells whose responses to cytokine-mediated stimulation depend on specific transcription factors (22, 38). Obtaining a deeper mechanistic understanding of the signaling mechanisms by which DCs control T cell responses, such as the BTLA-HVEM-CD5 axis described here, is necessary for the development of new immunotherapies for the treatment of cancer, autoimmunity, and infection.

## Author Contributions

All authors listed have made a substantial, direct and intellectual contribution to the work, and approved it for publication.

### Conflict of Interest Statement

The authors declare that the research was conducted in the absence of any commercial or financial relationships that could be construed as a potential conflict of interest.
